# Dietary intake of professional Australian football athletes surrounding body composition assessment

**DOI:** 10.1186/s12970-018-0248-5

**Published:** 2018-09-14

**Authors:** Sarah Louise Jenner, Gina Trakman, Aaron Coutts, Thomas Kempton, Samuel Ryan, Adrienne Forsyth, Regina Belski

**Affiliations:** 1 Department of Rehabilitation, Nutrition and Sport, La Trobe University, Bundoora, VIC Australia; 2 Carlton Football Club, Carlton, VIC Australia; 3 Human Performance Research Centre, University of Technology, Sydney, NSW Australia; 4 School of Health Sciences, Swinburne University of Technology, Hawthorn, VIC Australia

**Keywords:** Sports nutrition, Nutrition knowledge, Diet quality, Team-based sport

## Abstract

**Background:**

Sports Dietitians aim to assist in improving performance by developing nutrition knowledge (NK), enhancing dietary intake and optimising body composition of athletes. In a high-pressure environment, it is important to identify factors that may compromise an athlete’s nutrition status. Body composition assessments are regularly undertaken in sport to provide feedback on training adaptions; however, no research has explored the impact of these assessments on the dietary intake of professional athletes.

**Methods:**

This cross-sectional study assessed dietary intake (7-day food diary), nutrition knowledge (Nutrition for Sport Knowledge Questionnaire) and body composition (Dual-energy X-ray absorptiometry) of 46 professional male Australian football (AFL) athletes during a 2017 pre-season training week (7 days) where body composition assessments were undertaken. Dietary intake was assessed against International Olympic Committee recommendations for professional athletes.

**Results:**

Overall, no athlete met dietary their recommended energy intake (15 ± 1.1 vs. 9.1 ± 1.8 MJ, respectively) or carbohydrate recommendations (6–10 vs. 2.4 ± 0.9 g·kg-1·day-1). Only 54% met protein recommendations. Secondary analyses demonstrated significant associations between education status and energy intake (*P* < 0.04) and vegetable intake (*P* < 0.03), with higher levels of education being associated with higher intakes. A moderately positive association was observed between NK scores and meeting estimated energy requirements (*r* = 0.33, *P* = 0.03). NK scores were also positively associated with protein (*r* = 0.35, *P* = 0.02), fibre (*r* = 0.51, *P* = 0.001) and calcium intakes (*r* = 0.43, *P* = 0.004).

**Conclusions:**

This research identified that the dietary intake of professional AFL athletes during a pre-season training week where body composition assessments were undertaken did not meet current recommendations. Several factors may influence the dietary intake of AFL athletes, including lower education levels, poor NK and dietary intake restriction surrounding body composition assessment. Athletes may require support to continue with performance-based nutrition plans in periods surrounding body composition assessment.

## Background

Australian football (AF) is an intermittent high intensity team sport with intense periods of play, interspersed with periods of recovery [1]. At the professional level, Australian football (AFL) athletes train over a season that is divided into pre-season (~ 4 months) and competition (~ 6 months) phases. Training demands are substantial with athletes covering up to 30 km of total distance during a typical pre-season week, with a modified training volume performed in-season with the addition of match-play [2]. Moreover, athletes complete other training modalities (i.e. weights, cross training) to complement these running volumes in preparation for match demands. For a professional AFL athlete to meet training and performance demands and achieve body composition goals, energy and macronutrient intakes must reflect training and competition loads [3]. Therefore, dietitians have increasingly become recognized as an integral part of high performance teams. Their role is to promote optimal nutrition and to support, direct and teach sustainable nutrition practices to athletes in accordance with their training loads and body composition goals. Advice provided by accredited dietitians is guided by evidence-based recommendations that focus on periodization of energy, macronutrient and fluid intake, according to individual characteristics and body composition goals [4, 5].

Due to the high intensity nature of AF training and match play; adequate energy intake is required to maintain lean muscle mass, refuel energy stores and promote optimal performance [6]. However, research has reported that athletes across a range of team sports consistently fail to meet energy and carbohydrate recommendations [3, 6, 7]. The difficulties athletes experience when trying to consume a well-balanced diet and meet energy recommendations have also been reported [8, 9]. Many individual and environmental factors including knowledge and skills, peers and team culture, time constraints, finances and access to healthy food have been found to influence the dietary intake of athletes [6, 8, 10–12].

Nutrition knowledge (NK) is also thought to influence the dietary intake of individuals [11]. General knowledge behaviour models suggest that if an individual is aware of the benefits of nutrition they are more likely to make informed decisions regarding their health [13]. The link between NK and dietary behaviours has been examined in previous research, with weak to moderate positive associations reported in a variety of athletes [6, 7, 11]. While this research has underlined the importance of athlete NK and subsequent behaviour, most studies have only observed small samples of team sport athletes and used NK assessments that do not correspond with current IOC intake recommendations [14].

Coaches and high performance staff often assign athletes body composition goals such as reducing body fat and increasing lean muscle mass to develop a physique that can tolerate training loads and match demands [3]. Although body composition goals are present in professional sport, no research has established the impact of these goals on adequate intake. These goals are likely to influence the dietary intake of athletes, particularly during times of body composition assessment. However, in efforts to achieve body composition goals, restriction of dietary intake may occur and in times of high training loads may impair recovery, increase the likelihood of injury or illness and decrease overall performance (i.e. symptoms of overtraining) [8, 15, 16]. Body composition measurements may be collected during different training phases, so it is important to explore the extent to which body composition goals influence dietary intake, to inform strategies to support athletes to maintain adequate dietary intake required for performance.

Our primary aim was to examine the energy and macronutrient intakes of AFL athletes during a pre-season training week where body composition assessments were undertaken. Secondary aims included assessing intake of other nutrients and athletes’ NK as well as relationships between intake and factors including: age, playing experience and education status. We hypothesised that athletes were likely to have inadequate dietary intakes surrounding body composition assessment.

## Methods

### Participants

Athletes were recruited from one club competing in the AFL by the sports dietitian. Athlete characteristics (i.e. age and playing experience) and body composition data are reported in Table 1. Body composition (i.e. body fat percentage (%BF), fat free mass (FFM), height and body mass) was assessed using gold standard measure Dual-energy X-ray absorptiometry (DXA), following a method previously described elsewhere [3, 17].

**Table 1:** Participant characteristics

Characteristics	Mean ± SD
Age (years)	24.2 ± 4.0
Professional Experience (years)	5.5 ± 3.1
Height (cm)	188.4 ± 8.5
Body mass (kg)	86.3 ± 9.4
Body fat (%)	10.8 ± 2.3
Fat free mass (kg)	73.9 ± 9.1

### Dietary intake

Dietary intake was assessed using 7-day estimated food diaries collected during the pre-season phase of the season where body composition assessment was taking place. A 7-day food diary was selected as this time period captures a typical training micro-cycle in this athlete cohort, which includes three main skills sessions and all other training modalities [2]. Specifically, the observation period included three main training days (containing one skills training session [60–70 min] and one weights session [50 min] per day), one light training day (one short skills training session [40 min], one short weights session [30 min] per day) and three recovery days (no scheduled activity). Data was collected using the ‘Easy Diet Diary’ App (*n* = 41), ‘WhatsApp’ (*n* = 4) and one written food diary. The ‘Easy Diet Diary’ App was selected as it uses a database of Australian foods, has been validated as a method of assessing dietary intake in groups and provides a more convenient way of recording dietary intake than conventional written methods [18, 19]. The application ‘WhatsApp’ was used for four participants who could not access ‘Easy Diet Diary’ due to logistical issues. Food diaries estimated intake of all foods, fluids and supplements consumed on these days. Dietary intake data was analysed using a nutrition composition software program (Foodworks Version 8, Xyris, Queensland). Average daily energy and macronutrient intakes were analysed against current American College of Sports Medicine (ACSM) and International Olympic Committee (IOC) recommendations [4, 20, 21]. Individual energy requirements were estimated using the Harris-Benedict equation, using activity factors that reflected training loads [22]. Activity factors were categorised according to main, light and recovery training days (i.e. main: 1.75, light: 1.55 and recovery: 1.375). Intake was assessed in terms of food groups, calcium and fibre content relative to current Australian Guide to Healthy Eating (AGHE) and Nutrient Reference Values (NRVs) respectively [23, 24]. Under-reporting of energy intake was assessed using Goldberg thresholds [25]. A ratio of reported energy intake (EI) and basal metabolic rate (BMR) was calculated and compared to the cut-off limits of 0.9. Under-reporters were additionally evaluated against body composition goals.

### Nutrition knowledge

NK was assessed using the previously validated Nutrition for Sport Knowledge Questionnaire (NSKQ) [26]. The demographic section of the NSKQ asks about athlete characteristics (i.e. age and playing experience). The NSKQ contains 89 knowledge questions that assess various principles of sports nutrition. It is divided into six NK sub-sections; Weight Management (*n* = 13), Macronutrients (*n* = 30), Micronutrients (*n* = 13), Sports Nutrition principles (*n* = 13), Supplements (*n* = 12) and Alcohol (*n* = 8). Knowledge scores were calculated according to the NSKQ protocol [26]. Athletes were instructed to select agree/disagree or choose the correct answer from 4 to 5 possible options; they were told to select ‘not sure’ when appropriate. Overall performance in the NSKQ was assessed using the scoring system of; “poor” knowledge (0–49%), “average” knowledge (50–65%), “good” knowledge (66–75%) and “excellent” knowledge (75–100%) [26]. Participants were asked to complete the questionnaire using their own knowledge without the use of other resources (i.e. internet, peers etc.). All questionnaires were completed within a specified period of 7 days, consistent among individuals, in conjunction with the collection of dietary intake (i.e. 7-day food diary) and body composition data. Responses to an additional 19 investigator-designed questions regarding the participants’ views on current dietetic support, preferred means of education, self-efficacy of cooking skills and nutrition habits (i.e. portion sizes and periodization) were also collected. Responses to these additional questions were not included as a part of the overall knowledge score.

### Statistical analysis

Statistical analysis was conducted using IBM SPSS Statistics for Windows Version 24.0 (IBM Corp, Armonk, New York, USA, 2013). Statistical significance was set at *p* < 0.05. Shapiro-Wilk test for normality was used to assess if the data was normally distributed. Data was presented as means, standard deviations (±) and percentage of energy and macronutrient requirements (%) met, unless otherwise specified. Independent and paired sample t tests and ANOVA were used to assess significance between different groups (i.e. age, playing experience, education status). The proportion of participants meeting dietary intake recommendations for energy, carbohydrate, protein, fat, fibre, calcium and food groups (i.e. fruit and vegetable) was reported (Table 2). Dietary intake was then analysed in tertiles (i.e. low, medium, high intakes) against NK, age (years), playing experience (years) and education status, using a Chi-square test for independence.

**Table 2:** Total Energy, Macronutrient, Micronutrient (Calcium) and Food group intakes (mean ± SD) collected via a 7-day food diary

	Recommended Intake	Average per day (mean ± SD)	Number of Participants meeting recommendations (%, n)
Energy (MJ)	~ 15 MJ	9.1 ± 1.8	0 (*n* = 0)
Carbohydrate (g·kg^− 1^·day^− 1^)	6–10 g·kg^− 1^·day^− 1^	2.4 ± 0.8	0 (*n* = 0)
Protein (g·kg^− 1^·day^− 1^)	1.2–2.0 g·kg^− 1^·day^− 1^	1.8 ± 0.4	54 (*n* = 25)
Fat (g·kg^−1^·day^− 1^)	20–35% total energy	0.9 ± 0.3	91 (*n* = 42)
Fruit (serves)	2 serves per day	1.0 ± 0.8	91 (*n* = 42)
Vegetable (serves)	5 serves per day	4.3 ± 1.7	70 (*n* = 32)
Calcium (mg)	1000 mg-day^− 1^	952 ± 287	44 (*n* = 20)
Fibre (g)	30 g-day^− 1^	27.0 ± 7.6	37 (*n* = 17)

Energy and fat recommended intake as per American College of Sports Medicine (ACSM) [4]

Carbohydrate and protein recommended intake as per International Olympic Committee (IOC) [18]

Calcium and Fibre recommended as per Australian Nutrient Reference Values [21]

Fruit and Vegetable recommended intake as per AGHE recommendations [22]

Pearson’s correlation coefficient was used to describe the correlation between NK (i.e. overall score and subsection scores) and diet quality (i.e. % met total energy, macronutrients, fibre, calcium and food groups). They were interpreted according to correlation size (small: *r* = 0.10–0.29, moderate: *r* = 0.30–0.49 and large: *r* = 0.50–1.0) [27, 28].

## Results

### Dietary intake and body composition

None of the participants met current recommendations for energy and carbohydrate intake and only 54% met protein recommendations (Table 2). The majority also failed to meet calcium and fibre recommendations (Table 2). The majority (78%) of participants’ EI:BMR ratios were calculated as being below the Goldberg cut-off limit of 0.9 for a plausible intake. Participants who were not classified as under-reporters had body composition goals that aimed to increase total mass. Periodization of macronutrients such as carbohydrate was found to be inconsistent with recommendations, with food diaries demonstrating that the highest carbohydrate intake was found on light training days, followed by main training and recovery days respectively. On main training days, refined carbohydrate sources (i.e. processed and high GI carbohydrate foods) made up 60% ± 20% of total carbohydrate intake.

### Dietary intake and other factors

Relationships between meeting and not meeting recommendations for intake of total energy, carbohydrate, protein, fat, fibre, calcium and food groups (i.e. fruit and vegetable) and education status, level of playing experience and age were explored. Significant positive associations were found between education status and energy intake (*P* = 0.037) and vegetable intake (*P* = 0.028). There were no significant relationships found between education status and carbohydrate, protein, fat, fibre, calcium and fruit intake. No significant relationships were found between measures of dietary intake and age or level of playing experience.

### Nutrition knowledge

The response rate for NSKQ was 100%. The mean NK score for participants was 41 ± 13 out of a possible 89 points (46% mean score). The data was normally distributed. While overall NK was poor, participants performed best in macronutrient, weight management and sports nutrition sections (Table 3). A detailed description of professional and recreational AF players’ NK, assessed using the NSKQ are reported elsewhere [29].

**Table 3:** Participant’s Nutrition for Sport Knowledge Questionnaire overall and subsection scores

Section/Subsection (total items)	Min	Max	Median	Mean ± SD	Mean % Accuracy	Performance in the NSKQ
Weight Management (13)	1	13	6	6.4 ± 2.4	49	Poor
Macronutrients (30)	4	29	17	17.2 ± 5.1	58	Average
Micronutrients (13)	0	10	6	5.1 ± 2.5	39	Poor
Sports Nutrition (13)	1	11	6	6.1 ± 2.9	47	Poor
Supplements (12)	0	8	3	3.3 ± 1.9	28	Poor
Alcohol (8)	0	7	5	4.2 ± 1.8	53	Average
Overall Total Score (89)	9	70	42	41 ± 13	46	Poor

NSKQ scoring; Poor (0–49%), Average (50–65%), Above average (66–75%), Excellent (75–100%) [24]

Participants reported a high level of confidence in performing food related behaviours such as cooking a healthy meal (86.0 ± 1.2%). All participants responded that they sought nutrition advice and information from a dietitian (100%, *n* = 46) followed by teammates (80%, *n* = 37) and coach or trainer (41%, *n* = 19). There was a moderate positive association between NK scores and meeting estimated energy requirements (*r* = 0.325, *P* = 0.031). NK scores were also positively associated with protein (*r* = 0.348, *P* = 0.021), fibre (*r* = 0.510, *P* = 0.001) and calcium intakes (*r* = 0.428, *P* = 0.004).

## Discussion

### General findings

The aim of this study was to assess the dietary intake of professional AFL athletes during a pre-season training week where body composition assessments were undertaken. The main findings of the current study were (1) none of the AFL athletes met recommendations for energy or carbohydrate, (2) there was a moderate, positive association between NK and meeting energy requirements, (3) on average athletes had poor nutrition knowledge and (4) higher levels of education were associated with higher intakes of energy and vegetables. This supported our hypothesis that athletes would have inadequate dietary intakes surrounding body composition assessment.

### Effects of body composition goals on dietary intake

When data was explored in tertiles (i.e. low, medium and high) of energy intake, we found that athletes who had a greater energy intake reported a higher education status. Research by Johansson et al. explored under and over reporting of dietary intake in a non-athlete population and found a positive relationship between energy intake and education status [30]. Collectively, this suggests that education status may positively influence energy intake. Nonetheless, while education status improved energy intake in this cohort, 100% of athletes failed to meet energy intake recommendations [4, 20, 21]. Future research may explore the efficacy of different energy intake education approaches and other factors pertaining to energy intake in these athletes. However, although 78% of participants’ EI:BMR were calculated as being below the Goldberg cut-off of limit of 0.9 for a plausible intake, it is recognised that a lower than expected EI:BMR value may not simply be “under-reporting” but may instead indicate dieting or unusually low consumption during this training week. In this study, reports from participants suggest that whilst they did not meet energy and macronutrient recommendations, the food diaries are likely to be representative of actual dietary intake during that time period and low intakes are not due to under-reporting. Athletes are faced with external influences that may challenge diet adequacy and recovery. Body composition goals and pressures for athletes to make weight are a widely researched topic in professional sport [31, 32]. In particular, research has suggested that when body composition goals are present (i.e. required to lose fat mass or maintain a lean physique) there is usually an energy deficit and macronutrients required for refuelling and recovery are reduced [15]. In this study, food diaries were recorded during a time when the body composition of the AFL athletes was being assessed via DEXA scan. It is possible there was an intentional restriction of energy and carbohydrate intake as a result of aiming to meet the target body composition goals set for athletes, which was reflected in food records. This is supported by research that reported a desire for weight reduction as an explanation for failing to meet energy intake guidelines [30]. Taken together, our findings and those of others may indicate that psychological factors not quantified in this study may contribute to energy intake. To prevent training adaptions and recovery being compromised, it is important to promote realistic body composition goals to athletes. Future studies may use qualitative methods to explore the reasons for athletes not meeting energy and macronutrient recommendations in the time prior to a body composition assessment, and to highlight periods where under-fuelling may be present. This information may also provide a greater insight into the scheduling of body composition assessments in professional AFL programs. To promote a diet that supports performance and decrease the risk of injury, DXA scans may need to be avoided around key training sessions and game days.

### Other factors affecting energy and carbohydrate intake

Fluctuations in dietary intake that occur over a training week can be a result of several factors including hormonal influences on appetite regulation and time constraints within training schedules. Self-reported energy intake showed a level of periodization, with the largest energy intake reported on main training days followed by light and recovery days respectively. Similarly, participants had a good level of understanding of sport nutrition principles regarding periodization of carbohydrates and energy availability. Indeed, most participants (59%) reported that ‘periodizing energy and carbohydrate intake (i.e. high versus low days)’ was good practice for body composition management and performance [33]. Despite good knowledge of energy and carbohydrate periodization principles, total intake did not meet recommendations. Maintaining a diet that is energy and macronutrient deficient can increase the risk of illness and injury, promote weight loss (including losses to lean muscle mass) and alter performance outcomes [4]. Reasons why athletes may not consume adequate nutrients and energy during weeks of high training loads have previously been explored [8, 9]. In particular, physiological factors such as hormones may affect an athlete’s ability to consume the right amount of nutrients and energy required on main training days. Research has explored the influence of high intensity training on changes to appetite hormones and found that hormones such as ghrelin, glucagon-like peptide-1 (GLP-1), pancreatic polypeptide (PP) and peptide YY (PYY) may contribute to suppressed hunger post physical activity [9]. Furthermore, logistical issues such as time constraints on main training days may have additionally limited participants’ ability to consume adequate nutrition on main training days. Indeed, athletes appeared to choose convenient, energy dense snacks and refined carbohydrate sources on main training days. The potential provision of meals in this professional setting may positively support dietary intake over a training week. Future research may investigate whether the provision of meals in professional settings with the inclusion of flexible meal times in training schedules to accommodate those that experience supressed appetite post training can have a positive influence on athletes’ dietary intake over a training week.

### Carbohydrate intake

This study demonstrated that on average, AFL athletes’ dietary carbohydrate intake was relatively low in comparison to requirements based on training loads [3, 6]. This corresponds with previous research that reported dietary intakes of Australian footballers to fall below carbohydrate recommendations [3, 6]. Mean dietary carbohydrate intakes (2.4 ± 0.7 g·kg^− 1^·day^− 1^) fell below the lower range of sports nutrition guidelines advocated by the IOC (3–12 g·kg^− 1^·day^− 1^) [4, 34]. Carbohydrate is a key fuel used for high-intensity aerobic based sports like Australian football and plays a vital role as a muscle substrate for performance and recovery [35]. Low carbohydrate availability can lead to muscle glycogen depletion and subsequent fatigue, impaired concentration and decreased physical output [34, 36].

Research has suggested that carbohydrate intake is often the main macronutrient restricted by individuals to meet body composition goals [15]. Athletes had overall sound knowledge in the NSKQ macronutrients subsection, however it was found that knowledge regarding carbohydrate recommendations was poor. Therefore, poor NK may contribute to athletes having under-consumed carbohydrate. A large proportion of participants (41%) were ‘Not Sure’ regarding carbohydrate recommendations for athletes undertaking moderate to high intensity physical activity and only 24% of participants correctly identified carbohydrate recommendations. The present results are consistent with previous research, which found that 53% of collegiate athletes, when asked to identify carbohydrate recommendations, selected values that fell below carbohydrate recommendations advocated by sports nutrition experts [37, 38]. Previous findings show athletes have a misconception regarding carbohydrate availability (i.e. carbohydrate intake is related to body fat gains) and taken together the results of this study suggests that there is a knowledge gap regarding the importance of carbohydrate as a main fuel. It is important to promote individualised carbohydrate recommendations according to training requirements to support body composition goals and training adaptions of athletes. Further education that highlights the important role of carbohydrate in fuelling training and competition may be required, in addition to tailored advice to improve carbohydrate intake.

### Protein intake

Self-reported protein intakes in the present study were equal to or in excess of current recommendations for football and power sports [39]. Current recommendations suggest an absolute dose of 20–40 g per serve [39]. Indeed, 30% correctly responded to the statement ‘*Protein absorption in a single sitting is limited’.* Despite this, only 15% of participants reported that their usual serving size aligned with this recommended serve. It has been suggested that when energy availability is low (due to energy restriction aiming for weight loss), protein intake may be increased, to preserve muscle mass [40]. Due to the overall low energy intakes reported by athletes, excess protein ingestion may have been required to meet training demands. Extensive media attention regarding high protein, low carbohydrate diets may have additionally negatively influenced knowledge–creating an uncertainty regarding the distribution of macronutrients in the diet [10]. Future research could examine the scope of effect that media messages promoting low carbohydrate, high protein diets for weight loss, has on individuals’ dietary behaviours. Likewise, providing information regarding an athlete’s individual protein requirement and linking this information with education tools that focus on portion sizes of protein (e.g. serving size plates, visual portion guides) may help guide informed decisions about protein and simultaneously promote carbohydrate intakes that are better aligned with current guidelines.

### Nutrition education and dietetic support

Athletes were asked to give feedback regarding current dietetic support. Participants ranked individual consults as the most useful means of obtaining nutrition advice, in contrast to group presentations that were ranked as least useful. Given the findings of this study and the suitability of practical group based education in professional sport, these programs may need to include a more individualised approach that includes a greater focus on providing evidence-based information regarding energy and macronutrient requirements. Education that links dietary intake to performance may act as a motivator for behaviour change and limit the impact of other external factors, such as body composition assessments, on intake. Future research may investigate the efficacy of current practical nutrition education strategies such as cooking programs, in order to determine their effectiveness with the professional athlete cohort.

### Limitations

While this study aimed to assess the dietary intake of professional AFL athletes, it possesses several limitations. Firstly, the study sample included only one professional AFL team (*n* = 46) therefore the findings may reflect the individual characteristics and demographics of the group observed. Moreover, time constraints meant that only cross-sectional data could be collected. In addition to this, weighed food diaries are considered gold standard when assessing dietary intake in athletes; however, due to time and logistical constraints estimated food diaries were deemed the most appropriate method for use in this study.

Under-reporting is a well-documented limitation when using diet-recall methodologies to report nutrient intakes, especially in healthy cohorts [41]. Due to technological issues, 4 of the 46 participants in this study used ‘WhatsApp’ to report their dietary intake, as opposed to the vast majority of participants (*n* = 41) who used ‘Easy Diet Diary’. Those participants using ‘WhatsApp’ sent photos of their meals to the dietitian, who estimated portions and nutrient intake from this information, possibly resulting in the inaccurate measurement of dietary intake.

## Conclusion

This research provides insight into the dietary intake of AFL athletes during a pre-season training week where body composition assessments were undertaken. Our findings demonstrated that overall dietary intake did not meet recommendations. None of the athletes who participated in the study met energy or carbohydrate recommendations and only half met protein recommendations. Higher levels of education were associated with higher intakes of energy and vegetables and NK scores were also positively associated with meeting estimated energy requirements and protein, fibre and calcium intakes, demonstrating that several factors may influence the dietary intake of AFL athletes, including lower education levels, poor NK and dietary intake restriction surrounding body composition assessment. Athletes may require support to continue with performance-based nutrition plans in periods surrounding body composition assessment. We recommend the inclusion of individually tailored education programs to highlight the importance of dietary intake on body composition and performance, in addition to practical strategies to achieve recommended dietary intake.

## Abbreviations

AF: Australian Football
AFL: Australian Football League
DXA: Dual energy X-ray absorptiometry
NK: Nutrition Knowledge
NSKQ: Nutrition for Sport Knowledge Questionnaire
